# Personality Change Through Arts Education: A Review and Call for Further Research

**DOI:** 10.1177/1745691621991852

**Published:** 2021-07-20

**Authors:** Michael P. Grosz, Julia M. Lemp, Beatrice Rammstedt, Clemens M. Lechner

**Affiliations:** 1 Department of Psychology, University of Münster; 2 Heidelberg Institute of Global Health (HIGH), Medical Faculty and University Hospital, University of Heidelberg; 3 Department of Survey Design and Methodology, GESIS – Leibniz Institute for the Social Sciences, Mannheim

**Keywords:** personality change, personality development, arts interventions, arts education, self-esteem

## Abstract

Education involving active engagement in the arts, herein called *arts education*, is often believed to foster the development of desirable personality traits and skills in children and adolescents. Yet the impact of arts education on personality development has rarely been systematically investigated. In the current article, we reviewed the literature on personality change through arts education. We identified 36 suitable experimental and quasi-experimental studies. Evidence from these studies tentatively suggests that arts-education programs can foster personality traits such as extraversion and conscientiousness but not self-esteem. In addition, the effects of arts education appeared to be stronger in early and middle childhood than in preadolescence and early adolescence. However, the evidence for the effectiveness of arts education was very limited among the few included true experiments. Furthermore, the reviewed studies were heterogenous and subject to content-related, methodological, and statistical limitations. Thus, the current evidence base is inconclusive as to the effects of arts education on personality development. By identifying potential effects of arts education and limitations of past research, our review serves as a call for more research and guidepost for future studies on arts education and personality change.

An individual’s personality is linked to many important life outcomes. For example, personality traits are associated with academic performance, occupational attainment, and health after controlling for cognitive abilities (e.g., Almlund et al., 2011; Goodman et al., 2015; Lechner et al., 2017; Moffitt et al., 2011; Poropat, 2009; Roberts et al., 2007). It is also often theorized that personality traits such as conscientiousness affect outcomes such as health (e.g., Friedman et al., 2014; Shanahan et al., 2014), although experimental and nonexperimental research that explicitly estimates causal effects of personality traits on life outcomes is rare (e.g., Asendorpf et al., 2016; Grosz et al., 2020; Margolis & Lyubomirsky, 2020). Furthermore, personality changes throughout the entire life course (for reviews, see, e.g., Orth et al., 2018; Roberts et al., 2006; Soto & Tackett, 2015; Specht et al., 2014) and can be changed through interventions (for a review, see Roberts et al., 2017). On the basis of this literature, many researchers and practitioners believe that personality change may offer an attractive gateway for improving individual life outcomes and public welfare (e.g., Bleidorn et al., 2019; Sánchez Puerta et al., 2016). Accordingly, researchers, practitioners, and policymakers wonder how desirable personality characteristics—also called noncognitive skills, socioemotional skills, or soft skills—can be fostered (e.g., Alan & Ertac, 2018; Bleidorn et al., 2019; OECD, 2015; Sánchez Puerta et al., 2016).

Several researchers have proposed that the development of desirable personality features is fostered by artistic activities such as acting in plays and playing music (e.g., Aspin, 2000; Bamford, 2006). The arts are frequently believed to foster, for example, personality characteristics in the domains of agreeableness and extraversion because many artistic activities and performances require teamwork, negotiating, communication, and expressiveness, as well as the ability and willingness to lead and be led (e.g., Aspin, 2000; Winner et al., 2013). The believe that artistic activities foster desirable personality traits might even be a reason why the arts are highly prevalent in the curriculum of many educational institutions and why governments around the globe invest in education involving active engagement in the arts, henceforth called *arts education*.

Are hopes in arts education as a means through which to foster desirable personality traits justified? There is a considerable body of work on the effects of arts education, especially music training, on cognitive abilities and academic performance. This research shows only limited evidence for the effectiveness of arts education, especially when considering experimental studies with active controls (for reviews, see Cooper, 2020; Sala & Gobet, 2020; Swaminathan & Schellenberg, 2014). In contrast to the effects of arts education on cognitive abilities, the effects of arts education on personality have received little attention. This gap poses the risk that current efforts by schools and other educational institutions might be ineffective. The lack of research on the effectiveness of arts education is also unfortunate from the perspective of research on personality. Because studies on arts education often involve interventions, such studies might indicate the kinds of environmental factors that spur certain kinds of personality change. Such causal links are difficult to identify by the noninterventional (i.e., observational) designs that prevail in research on personality development. The current study offers a review and synthesis of experimental and quasi-experimental studies on how arts education interventions affect the development of the Big Five personality traits (extraversion, emotional stability, agreeableness, conscientiousness, and openness to experience) and self-esteem. We focused on the Big Five and self-esteem because the Big Five framework is currently the most widely used model of personality, whereas self-esteem is the most widely studied individual-difference construct outside of the Big Five framework.

In the course of the review, we regard increases in the domains of extraversion, agreeableness, openness, emotional stability, conscientiousness, and self-esteem as desirable because (a) people on average desire to change their own personality in these directions (e.g., Hudson & Fraley, 2015; Hudson & Roberts, 2014) and (b) these traits are theorized to cause desirable life outcomes. For example, conscientiousness is believed to increase health and longevity via health-related behaviors (e.g., Friedman et al., 2014; Shanahan et al., 2014). Extraversion is believed to increase well-being via social behaviors and relationships (e.g., Lee et al., 2008; Margolis & Lyubomirsky, 2020; Steel et al., 2008). Self-esteem is believed to be a protective factor against developing depressive symptoms (e.g., Beck, 1967; Masselink et al., 2018). If these theories are correct, then arts education that fosters traits such as conscientiousness, extraversion, and self-esteem might lead to desirable outcomes such as health, well-being, and fewer depressive symptoms. That said, we would like to point out that increases in extraversion, agreeableness, openness, emotional stability, conscientiousness, and self-esteem might not be (equally) desirable or adaptive for everyone (e.g., Buss, 2009; Loewenstein, 2018).

## The Malleability of the Big Five Personality Traits Through Interventions

The belief that arts education can foster personality development rests on the notion that personality is malleable. In line with this notion, the past decades of longitudinal observational studies have shown that mean-level and rank-order changes in the personality traits occur across the entire life span (e.g., Roberts et al., 2006; Soto & Tackett, 2015). Previous longitudinal research has also suggested that changes in personality traits can be driven by environmental influences (for reviews, see e.g., Specht et al., 2014; Wrzus & Roberts, 2017). Yet most longitudinal observational evidence is inconclusive regarding which experiences trigger personality change (e.g., Bleidorn et al., 2018; Denissen et al., 2019).

Intervention studies on personality traits are comparatively rare. Roberts et al. (2017) systematically reviewed studies on therapeutic interventions (e.g., to treat depression) and found a sizeable weighted average pretest–posttest effect size across the Big Five personality domains (*d_z_* = 0.37, 95% confidence interval [CI] = [0.33, 0.40]). That said, selection bias might have affected this effect size estimate because most of the reviewed studies were nonexperimental (i.e., assignment to the treatment and control conditions was nonrandom). Furthermore, publication bias might have been an issue. After correcting for small-study effects, the average effect size across all personality traits in the experimental studies was small (*d* = 0.13, 95% CI = [−0.10, 0.36]), although there was still a relatively large effect on emotional stability (*d* = 0.39, 95% CI = [0.07, 0.70]). Mindfulness training and cognitive training have also been found to be associated with changes in personality (Jackson et al., 2012; Krasner et al., 2009; but see also Sander et al., 2017). Furthermore, recent randomized controlled studies reported that educational interventions can increase grit (i.e., a personality trait from the conscientiousness family that is characterized by long-term persistence in goal pursuit; Alan et al., 2019; Alan & Ertac, 2018; see also, Bettinger et al., 2018). Taken together, these studies suggest that interventions have the potential to change personality traits, and such potential is a necessary (but not sufficient) condition for an effect of arts education on personality traits.

Most previous intervention and observational studies have focused on personality change in adulthood. This is unfortunate because interventions might be particularly effective in children and adolescents given that the average rank-order stability of personality traits steadily increases throughout the life span (e.g., Ferguson, 2010; Soto & Tackett, 2015). Furthermore, recent research has suggested that the structure of personality in childhood and adolescence is more similar to the structure of personality in adulthood than previously thought (e.g., Herzhoff et al., 2017; Soto, 2016; Soto & Tackett, 2015). Accordingly, an increasing number of observational studies and some intervention studies have been devoted to personality development in the first 2 decades of life (e.g., Alan et al., 2019; Alan & Ertac, 2018; Bettinger et al., 2018; Göllner et al., 2017). Yet there is still a distinct lack of intervention studies on personality change in childhood and adolescence. We think this lacuna could be addressed by interventional research on arts education and personality change because most arts-education studies are conducted with school-age children—the arts education is either part of their formal education or is an extracurricular activity.

## The Malleability of Self-Esteem Through Interventions

Mean-level and rank-order changes across the entire life span have been observed not only for personality traits but also for self-esteem (e.g., Orth et al., 2018; Trzesniewski et al., 2003). Yet in contrast to most studies on the development of the Big Five personality traits, many studies on self-esteem development have used experimental or quasi-experimental designs (for meta-analyses, see, e.g., Haney & Durlak, 1998; Liu et al., 2015; O’Mara et al., 2006). Furthermore, a large part of experimental and quasi-experimental research on self-esteem has focused on interventions during childhood and adolescence. For example, a meta-analysis found that, among 25 experimental studies, physical-activity interventions had a positive effect on children’s and adolescents’ self-esteem (Liu et al., 2015; see also Ekeland et al., 2005). Building on this research tradition, the current review will also investigate the existing literature on effects of arts-education interventions on self-esteem development in youth.

## How and Why Arts Education Might Change Personality

Several models of personality development propose that long-term change in personality traits occurs because of repeated short-term state processes (e.g., Geukes et al., 2018; Wrzus & Roberts, 2017). On the basis of this theoretical bedrock, we propose four ways how arts education might induce long-term personality trait changes.

First, three forms of arts education, drama, music, and dance, are usually social in nature (e.g., playing in a school band), and they evoke and demand friendly, collaborative, and outgoing behavioral tendencies. By repeatedly demanding and affording warm and expressive behavior, drama, music, and dance education might increase extraversion and agreeableness (i.e., the two personality domains that are most relevant for interpersonal behavior; see, e.g., DeYoung et al., 2013). For example, extraverted and agreeable behavior might be adaptive for making friends in extracurricular theater training or for getting help with challenging theater, music, or dance tasks. Drama, music, and dance activities might furthermore require the ability and willingness to lead and be led, as argued by Aspin (2000). Finally, drama, music, and dance activities demand the ability to place oneself in someone else’s shoes, and thus arts education might foster respect and understanding of others (e.g., Winner et al., 2013). As a result of all of these situational demands and opportunities for state expressions of extraversion and agreeableness, drama, music, and dance education might increase trait levels of extraversion and agreeableness in the long run.

Second, all kinds of arts education (drama, music, dance, and visual arts and crafts) might foster conscientiousness because arts trainings with their behavioral rules (e.g., memorizing and repeatedly practicing scripts and dance moves) might require a high level of discipline and self-control. Coinciding with this notion, research on the effects of homework and vocational training has suggested that demands for discipline and self-control can lead to increases in conscientiousness (Golle et al., 2018; Göllner et al., 2017).

Third, all kinds of arts education might lead to higher levels of openness to experience because an engagement with arts might reinforce several central aspects of the openness domain. It might stimulate interest and fascination for the arts (i.e., visual arts, music, dance, etc.), induce an appreciation of novel ideas and perspectives on the world, and stimulate philosophical thoughts and discussions. Openness is strongly associated with artistic activities, interests, and preferences (e.g., McCrae & Sutin, 2009; Schwaba et al., 2018). Although these associations might largely be due to selection effects, repeated engagement in arts activities might have also socialization effects on openness.

**Fig. 1.**
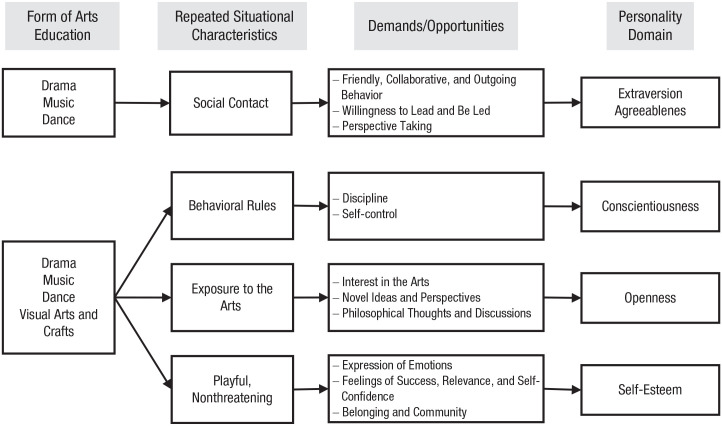
Four potential paths from arts education to personality trait change.

Fourth, all forms of arts education might foster self-esteem because arts activities can provide children with appealing and nonthreatening opportunities to express themselves; to feel successful, relevant, and self-confident; and to build a sense of belonging and community (e.g., Rickard et al., 2013; Winner et al., 2013). A summary of the four paths from arts education to personality trait change is depicted in Figure 1.^1^

## A Review of Empirical Studies on Arts Education and Personality Change

Taken as a whole, the literature suggests that personality traits and self-esteem are, in principle, malleable through clinical and nonclinical interventions. Moreover, several potential mechanisms might drive an effect of arts education on personality development. However, does empirical evidence exist for the proposed effects of arts education on personality? Winner et al. (2013) reviewed the effects of arts education on social skills. Yet they considered only a specific subset of personality measures as target outcomes, mostly from the domains of agreeableness, extraversion, and self-esteem. The current review updates and extends the review by Winner et al. Our review includes measures from all Big Five personality domains and self-esteem, and it covers not only articles published prior to 2013 but also articles published between 2013 and 2018 that were not covered in Winner et al. (2013). Furthermore, our review focuses exclusively on quasi-experimental and experimental studies. Finally, we used systematic and explicit methods in all stages of our review (i.e., scoping, searching, screening, eligibility, and reporting) so as to minimize subjectivity and bias and maximize transparency and replicability.

Our review adopts an approach similar to the one by Roberts et al. (2017). Roberts et al. reviewed the literature on the effects of therapeutic interventions, most of which were clinical studies, to address the lack of intervention studies on personality trait change in adulthood. The majority of the studies that they included did not explicitly focus on changing personality traits; rather, they incidentally measured personality traits or outcome variables that were essentially personality traits. Likewise, in the educational literature on arts interventions, there are a number of studies that often did not explicitly focus on changing personality traits but included outcome measures that were essentially measures of personality traits. That is, the measures used in these studies (a) conformed to the conventional definition of personality traits as relatively enduring patterns of thoughts, feelings, and behaviors; (b) referred to enduring *traits*, rather than only temporary states; (c) assessed to some degree one or more of the Big Five domains; and (d) comprised items that represented general patterns—as opposed to patterns specific to arts education—of thoughts, feelings, and behaviors (for details on the measures, see Tables S1 to S7). Furthermore, many studies in the educational literature have investigated the impact of arts education on measures of self-esteem or closely related measures (e.g., general self-concept).

Two content questions guided the current review. First, does arts education have effects on the personality development of children and adolescents? Second, is there empirical evidence for the proposed pathways from arts education to personality change (Fig. 1)? These two questions are of theoretical significance for personality and developmental psychology because the answers will help to identify the factors that drive personality change in youth, which will enhance our understanding of why and how interventions (or environmental factors in general) lead to personality development. In turn, this know-how will have far-reaching implications for educators and policymakers who are interested in using the arts to foster personality development.

Finally, a word on normative personality developments during childhood and adolescents is in order. First, the maturity principle proposes conscientiousness, emotional stability, and agreeableness increase with age (e.g., Brandes et al., 2020; Caspi et al., 2005). Second, the disruption hypothesis states that biological, social, and psychological changes lead to a setback in desirable personality traits (i.e., conscientiousness and agreeableness) during adolescence (e.g., Brandes et al., 2020; Soto & Tackett, 2015). Third, a recent meta-analysis indicates that mean levels of self-esteem increase from ages 4 to 11 and remain stable from ages 11 to 15 (Orth et al., 2018). Taken together, several normative trends seem to take place during childhood and adolescence. There is no consensus on the exact nature and timing of these trends. Thus, the current review focuses on arts-education studies with control group designs to disentangle normative changes in personality from changes induced by arts education.

## Method

The current review was exploratory in nature. At the outset of our review, we were not sure about the extent of the literature on arts education and personality change. Hence, our aim was not to test specific hypotheses, as is typically the case in a meta-analysis or systematic review. Our aim, rather, was to explore how much literature there is on the topic and what the features, main findings, and limitations of this literature are. Hence, we did not preregister a protocol or hypotheses.

### Literature search

#### Electronic database search

We used three word groups in the electronic database search. Word Group 1 included “personality” and synonymous or related terms that are commonly used in arts-education studies: Personality, Temperament, Socioemot*, Socio-emot*, Non-cogn*, Noncogn*, “Social skills,” “Personal skills,” “Life skills,” “Emotional skills,” “Soft skills.” Word Group 2 included concrete art forms and activities: Art, Arts, Music*, Danc*, Sing*, Theat*, Drama*, Opera, Fiction, Reading, Craft*, Sculpt*, Poet*, Extracurricular*, Afterschool, After-school. Word Group 3 included several umbrella terms that can be used as synonyms for arts education: “Cultural education,” “Cultural participation,” “Cultural literacy,” “Cultural capital,” “Cultural exposure,” “Cultural experience,” “Cultural consumption,” “Cultural exchange,” “Cultural activit*,” “Cultural visit,” “High culture,” “Highbrow.” In the full electronic database search, Word Group 1 was combined with either Word Group 2 (Search A) or Word Group 3 (Search B). We searched for the keywords in the Web of Science. The search mask allowed us to automatically remove duplicates from Search A in Search B. The search was first conducted in July 2018 and updated through September 2018. All studies that were electronically available until September 30, 2018, were included.

#### Other sources

In addition, we manually screened the references from the eligible articles identified in the electronic search, the references from some ineligible articles, and the references from the review by Winner et al. (2013) for relevant studies. Finally, eligible articles from exploratory searches were included.

### Study selection

All records identified from the electronic database search were screened on the basis of their titles and abstracts. Studies that were judged as potentially relevant after we reviewed the abstract were fully accessed. The question of whether to include a fully accessed article was independently addressed by two people (i.e., authors M. P. Grosz and J. Lemp). Conflicts were solved by refining the eligibility criteria and through discussions with author C. M. Lechner.

**Fig. 2.**
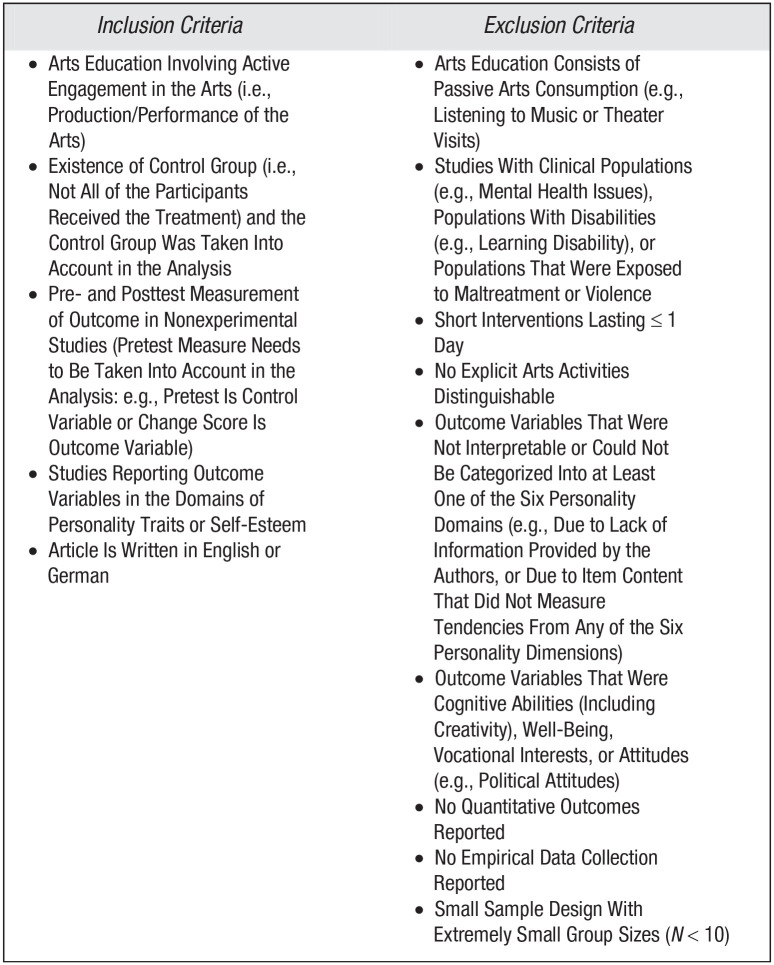
Inclusion and exclusion criteria.

As specified in the inclusion and exclusion criteria displayed in Figure 2, we included only studies that comprised arts-education interventions that required an active engagement in the arts (i.e., the production and performance of the arts). Passive consumption of the arts (e.g., listening to music) is ubiquitous, ill-defined, and would thus be difficult to investigate. Because our interest was in causal evidence, we included only studies that used experimental designs (randomized controlled trials) and quasi-experimental designs (i.e., pretest–posttest designs without the randomized assignment of individuals to experimental and control groups). For reasons outlined above, we included only studies with control group designs. Furthermore, among quasi-experimental studies, we included only those with pretest measures of the outcome variable because, when studies do not adjust for the pretest, it is difficult to disentangle socialization from selection.

We considered both studies on broad personality domains and studies on more specific cognitive, affective, and behavioral tendencies (i.e., personality facets or nuances from each of the six broader domains). This inclusive strategy is in line with recent research emphasizing that facets and nuances are valid and stable aspects of personality (e.g., Mõttus et al., 2017). Moreover, intervention studies typically target narrow and specific outcomes rather than broad traits. We did not include cognitive abilities (including creativity) and vocational interests because personality traits have often been distinguished from abilities and motivations (e.g., McAdams & Pals, 2006; Penke et al., 2007; Roberts & Wood, 2006). We did not include subjective well-being and behavioral intentions because these individual differences are less stable than personality traits. Finally, we did not include attitudes because attitudes are to a larger extent acquired through experience and are more object-oriented than personality traits. There were no restrictions on the year of publication, age, or the country of origin of the targeted population.

### Effect-size calculations

The majority of the studies did not report any effect sizes. To increase the comparability of the effects across studies, we therefore computed effect-size estimates from the reported summary and test statistics. In accordance with Roberts et al. (2017) and Lakens (2013), we manually computed two effect sizes: Cohen’s *d_z_* and Cohen’s *d_s_*. For studies that used a pretest–posttest design, we calculated Cohen’s *d_z_* directly from the *t* statistic for the pretest–posttest difference divided by the square root of the number of participants (see also Rosenthal, 1991):

(1) $$d_{z}=\frac{t}{\sqrt{n}}.$$

Whenever the *t* statistics was not reported, we calculated the *t* value by dividing the pretest–posttest difference by the standard deviation of the pretest, as was done by Roberts et al. (2017).^2^ For experimental studies, we additionally calculated Cohen’s *d_s_* whenever possible. We did so by dividing the mean postintervention difference between the treatment group (EG) and control group (CG) by their pooled standard deviation:

(2) $$d_{s}\;=\frac{{\bar{x}}_{\mathrm{EG}}-\;{\bar{x}}_{\mathrm{CG}}}{\sqrt{\frac{\left(n_{1}-1\right)SD_{1}^{2}+\left(n_{2}-1\right)SD_{2}^{2}}{n_{1}+n_{2}-2}}}.$$

Alternatively, we calculated Cohen’s *d_s_* from the *t* statistic for the postintervention difference:

(3) $$d_{s}\;=\;t\times \sqrt{\frac{1}{n_{1}}+\frac{1}{n_{2}}}.$$

Cohen’s *d_z_* refers to the standardized mean difference effect size for the difference between a personality variable at Time 1 and the same personality variable at Time 2 within the same group (i.e., either within the treatment group or within the control group). Cohen’s *d_s_* refers to the standardized mean difference on the personality variable between treatment and control groups within the same measurement occasion (i.e., at Time 2). We calculated *d_s_* only for experimental studies but not nonexperimental studies because in the latter, the difference between treatment and control group might be confounded by selection bias (i.e., selection into treatment).

We reversed the sign of the effect sizes for change in undesirable outcomes (e.g., internalizing problems) to ensure that the effect sizes were always positive when participants increased in extraversion, agreeableness, openness, emotional stability, conscientiousness, and self-esteem.

## Results and Discussion

Supporting tables and figures, details about the literature search, basic information about each study, effect size calculations, the data, and the R code for data analysis can be found at OSF: https://osf.io/yxqc7/.

### Overview of the included studies

The electronic database search resulted in 7,732 initial hits without duplicates. Screening the titles and abstracts of the hits reduced the number of articles to 134. Scrutinizing the full texts of the 134 articles resulted in 12 articles that met all of our eligibility criteria. Screening the reference lists of these 12 identified articles for relevant studies resulted in nine additional eligible articles. Screening the reference lists of some ineligible articles and of the past review by Winner et al. (2013) resulted in 11 additional articles. Finally, one article was identified in an exploratory search (for a flow diagram, see Fig. S1 at https://osf.io/69yz8/). In total, we thus included 33 articles that reported the results of 36 experimental and quasi-experimental studies containing 43 samples receiving arts education (for a list of articles that were excluded, see Table S8 at https://osf.io/z5xsm/). The number of samples is higher than the number of studies because several studies contained more than one treatment group. We assigned to each treatment group a unique sample ID.

In the following, we present aggregated information about the characteristics of the 33 articles and 36 studies (e.g., median age across studies). Disaggregated information about each study can be found in Tables 1 to 5. The average publication year was 2009 (range = 1984–2018). Most studies were conducted in North America (60%), followed by Europe (17%) and Australia (14%). Far fewer studies came from South America, Asia, or Africa (3% each). Most of the studies were conducted in school-age children. Accordingly, participants had a comparatively young median age of 9.50 years (*SD* = 4.06; range = 0.5–23 years). Twenty-nine studies used a quasi-experimental design (i.e., without random assignment to condition), and seven studies employed an experimental design (i.e., with random assignment to condition).

**Table 1.** Drama-Education Studies

Study	Intervention	Duration (weeks)	Outcome (measure)	Assessment	Design	*M* Age	*N* ^a^	Effect (test)	Effect size
Extraversion									
Catterall (2007)	Drama	24	Work effectively in group	Self-report	Q-EXP	12.5	155	+	—^b^
Goldstein et al. (2012), Study 1; Goldstein et al. (2013), Study 2	Acting (vs. visual arts)	43	Emotional expressivity – positive (BEQ)	Self-report	Q-EXP	8.5	68	n.s.	*d_z_* = 0.06 *d_s_* = 0.15
Gourgey et al. (1984)	Improvisational Dramatics (incl. playwriting)	20	Self-expression	Self-report	Q-EXP	10.5	158	+	—^b^
Nicolopoulou et al. (2015)	Story-acting practice	43	Self-assertion	Teacher-rated	EXP	3.5	60	n.s.	*d_z_* = −0.11 *d_s_* = 0.49
Schellenberg (2004)	Drama lessons	36	Adaptive social functioning (BASC)	Parent-rated	Q-EXP	6	132	+	*d_z_* = 0.69 *d_s_* = 0.57
Walsh-Bowers (1992)	Creative drama	14	Peer Cooperation (PIS)	Self-report	Q-EXP	11.5	104	n.s.	*d_z_* = 0.47 *d_s_* = 0.39
			Peer Conflict (PIS)	Self-report	Q-EXP	11.5	104	n.s.	*d_z_* = 0.50 *d_s_* = 0.24
Walsh-Bowers et al. (1999), Study 1	Creative drama	15	Peer Cooperation (PIS)	Self-report	Q-EXP	12.5	44	n.s.	*d_z_* = 0.62 *d_s_* = −0.04
			Peer Conflict (PIS)	Self-report	Q-EXP	12.5	44	n.s.	*d_z_* = 0.86 *d_s_* = 0.39
Walsh-Bowers et al. (1999), Study 2	Creative drama	15	Peer Cooperation (PIS)	Self-report	Q-EXP	12.5	75	n.s.	*d_z_* = 0.30 *d_s_* = −0.16
			Peer Conflict (PIS)	Self-report	Q-EXP	12.5	75	n.s.	*d_z_* = 0.37 *d_s_* = −0.14
Emotional stability									
Freeman et al. (2003)	Creative drama	18	Problem behavior SSRS	Teacher-rated	EXP	9	102	n.s.	*d_z_ =* 0.15 *d_s_* = −0.17
Goldstein et al. (2018)	Dramatic pretend play	8	Personal distress	Observer-rated	EXP	4	51	+	*d_z_* = −0.43 *d_s_* = −0.82
Goldstein et al. (2012), Study 1; Goldstein et al. (2013), Study 2	Acting (vs. visual arts)	43	Emotional expressivity – negative (BEQ)	Self-report	Q-EXP	8.5	68	n.s.	*d_z_* = 0.37 *d_s_* = −0.06
Metsäpelto et al. (2012)	Performing arts	156	Internalizing problems (TR-MPNI)	Teacher-rated	Q-EXP	9.5	217	n.s.	—^b^
Rickard et al. (2012), Study 2	Enhanced drama	20	Reynolds Child Depression Scale	Self-report	EXP	10.9	68	n.s.^c^	*d_z_* = 0.01 *d_s_* = −0.23
			Problem behavior (SSRS)	Parent-rated	EXP	10.9	68	n.s.^c^	*d_z_* = 0.30 *d_s_* = −0.61
Agreeableness									
Catterall (2007)	Drama	24	Work with others when disagreeing^d^	Self-report	Q-EXP	12.5	155	n.s.	—^b^
Goldstein et al. (2012), Study 1; Goldstein et al. (2013), Study 2	Acting (vs. visual arts)	43	Theory of mind (Faux Pas Test)	Objective	Q-EXP	8.5	68	n.s.	*d_z_* = 0.69 *d_s_* = −0.01
			Index of Empathy for Children (IECA)	Self-report	Q-EXP	8.5	68	*+*	*d_z_* = 0.61 *d_s_* = 0.73
Goldstein et al. (2012), Study 1; Goldstein et al. (2013), Study 2	Acting (vs. visual arts)	43	Basic Empathy Scale	Self-report	Q-EXP	14.4	48	n.s.	*d_z_* = 0.21 *d_s_* = 0.31
Goldstein et al. (2018)	Dramatic pretend play	8	Theory of mind	Objective	EXP	4	51	n.s.	—^b^
			Comforting (observational ratings)	Observer-rated	EXP	4	51	n.s.	*d_z_* = 0.00 *d_s_* = .025
			Helping (observational ratings)	Observer-rated	EXP	4	51	n.s.	*d_z_* = −0.22 *d_s_* = −0.21
			Social interaction – negative (SIOS)	Observer-rated	EXP	4	51	n.s.	*d_z_* = 0.62 *d_s_* = −0.60
			Emotion matching (Index of Empathy)	Observer-rated	EXP	4	51	—	*d_z_* = −0.22 *d_s_* = −0.31
			Altruism (dictator)	Objective	EXP	4	51	n.s.	*d_z_* = −0.09 *d_s_* = −0.11
Gourgey et al. (1984)	Improvisational Dramatics (including playwriting)	20	Trust	Self-report	Q-EXP	10.5	158	n.s.	—^b^
			Acceptance of others	Self-report	Q-EXP	10.5	158	+	—^b^
Nicolopoulou et al. (2015)	Story-acting practice	43	Disruption (Peer play Disruption; PIPPS)	Observer-rated	EXP	3.5	112	n.s.^e^	*d_z_* = 0.11 *d_s_* = 0.25
Rickard et al. (2012), Study 2	Enhanced drama	20	Aggression Questionnaire	Self-report	EXP	10.9	68	n.s.^c^	*d_z_* = −1.08 *d_s_* = −1.57
Conscientiousness									
Metsäpelto et al. (2012)	Performing arts	156	Working skills (i.e., persistence, concentration, and carefulness)	Teacher-rated	Q-EXP	9.5	217	+	—^b^
Nicolopoulou et al. (2015)	Story-acting practice	43	Self-inhibition (Kashiwagi teacher rating scale)	Teacher-rated	EXP	3.5	60	+	*d_z_* = 0.61 *d_s_* = 1.42
Self-esteem									
Freeman et al. (2003)	Creative drama	18	Self-concept (SSCS)	Self-report	EXP	9	91	n.s.	*d_z_* = 0.47 *d_s_* = −0.42
Gourgey et al. (1984)	Improvisational Dramatics (including playwriting)	20	Self-acceptance	Self-report	Q-EXP	10.5	158	n.s.	—^b^
Rickard et al. (2012), Study 1	Enhanced drama	22	Self-esteem (CFSEI-3)	Self-report	Q-EXP	12.7	111	n.s.	*d_z_* = −0.42
Rickard et al. (2012), Study 2	Enhanced drama	20	Self-esteem (RSE)	Self-report	EXP	10.9	68	n.s.^c^	*d_z_* = 0.00 *d_s_* = −0.55
Blended									
Freeman et al. (2003)	Creative drama	18	Social skills (SSRS)	Teacher-rated	EXP	9	102	n.s.	*d_z_ =* 0.27 *d_s_* = −0.27
Goldstein et al. (2018)	Dramatic pretend play	8	Social interaction – positive (SIOS)	Observer-rated	EXP	4	51	n.s.	*d_z_* = −0.69 *d_s_* = 0.83
Metsäpelto et al. (2012)	Performing arts	156	Adaptive behavior (TR-MPNI)	Teacher-rated	Q-EXP	9.5	217	n.s.	—^b^
			Externalizing problems (TR-MPNI)	Teacher-rated	Q-EXP	9.5	217	n.s.	—^b^
Nicolopoulou et al. (2015)	Story-acting practice	43	Interaction (Peer play cooperation; PIPPS)	Observer-rated	EXP	3.5	112	n.s.	*d_z_ =* 0.13 *d_s_* = 0.49
Rickard et al. (2012), Study 2	Enhanced drama	20	Social skills (SSRS)	Self-report	EXP	10.9	68	n.s.^c^	*d_z_* = 0.26 *d_s_ = −*0.22
				Parent-rated	EXP	10.9	68	n.s.	*d_z_* = 0.69 *d_s_ = −*0.05

Note: Cohen’s *d_z_* was calculated directly from the *t* statistic divided by the square root of the number of participants. Whenever the *t* statistic was not reported, we calculated the *t* value by dividing the pretest–posttest difference by the standard deviation of the pretest. For experimental studies, we additionally calculated Cohen’s *d_s_* whenever possible (for details, see the Method section). + = positive effect. BASC = Behavioral Assessment System for Children; BEQ = Berkeley Expressivity Questionnaire; CFSEI-3 = Culture Free Self-Esteem Inventories Third Edition; EXP = experimental design (randomized controlled trial); IECA = Empathy Index for Children and Adolescents; PIPPS = Penn Interactive Peer Play Scale; PIS = Peer Interaction Scale; Q-EXP = quasi-experimental design; RSE = Rosenberg Self-Esteem Scale; SIOS = Social Interaction Observation System; SSCS = Student Self-Concept Scale; SSRS = Social Skills Rating System; TR-MPNI = Multidimensional Peer Nomination Inventory, Teacher Rating Form.

a *N* refers to the sample sizes of the experimental and control groups combined. ^b^Effect size *d_z_* was not available and it could not be calculated from the results reported in the study. ^c^The reported two-way (Group × Time) mixed-model ANOVA included not only a music group and a control group but also a drama group. ^d^Measured with a self-developed scale with multiple items. ^e^There was no effect overall. Yet there was a significant Year × Condition interaction effect. The intervention was conducted in two randomized blocks (different years). For Year 1, the difference in peer play disruption between the experimental and control groups was in a desirable direction and was statistically significant.

**Table 2.** Music-Education Studies

Study	Intervention	Duration (weeks)	Outcome (measure)	Assessment	Design	*M* Age	*N* ^a^	Effect (test)	Effect size
Extraversion									
Gerry et al. (2012)	Active parent-infant music classes	36	Smiling and laughter	Parent-rated	EXP	0.5	34	+	—^b^ *d_s_* = 0.20
Schellenberg (2004)	Music lessons	36	Adaptive social functioning (BASC)	Parent-rated	Q-EXP	6	132	n.s.	—^b^
Emotional stability									
Gerry et al. (2012)	Active parent-infant music classes	36	Distress to limitations	Parent-rated	EXP	0.5	34	+	—^b^ *d_s_* = 1.22
			Distress to novel situations	Parent-rated	EXP	0.5	34	+	—^b^ *d_s_* = 1.62
Metsäpelto et al. (2012)	Music activities	156	Internalizing problems (TR-MPNI)	Teacher-rated	Q-EXP	9.5	177	n.s.	—^b^
Rickard et al. (2012), Study 2	Enhanced music lessons	20	Reynolds Child Depression Scale	Self-report	EXP	10.9	69	n.s.^c^	*d_z_* = 0.04 *d_s_* = 0.03
Roy et al. (2015)	School-band program	130	Optimism (IPIP)	Self-report	Q-EXP	13.5	31	n.s.	—^b^
			Prevention focus (GRFM)	Self-report	Q-EXP	13.5	31	n.s.	—^b^
Rickard et al. (2013; younger cohort)	Enhanced music lessons	104	Problem behavior (SSRS)	Teacher-rated	Q-EXP	6	97	n.s.	—^b^
Rickard et al. (2013; older cohort)	Enhanced music lessons	104	Problem behavior (SSRS)	Teacher-rated	Q-EXP	9	67	n.s.	—^b^
Rickard et al. (2012; Study 2)	Enhanced music lessons	20	Problem behavior (SSRS)	Parent-rated	EXP	10.9	69	n.s.	*d_z_* = 0.21 *d_s_* = −0.70
Agreeableness									
Kalliopuska et al. (1993)	Music-based empathy education program	12	Feshbach and Roe Empathy Slide Test	Objective	Q-EXP	6	27	+	*d_z_* = 2.89 *d_s_* = 1.65
			Prosociability (Weir and Duveen scale)	Teacher-rated	Q-EXP	6	30	+	—^b^ *d_s_* = 1.81
Rabinowitch et al. (2013)	Musical group interaction	31 (13 or 39)	Empathy (Matched faces)	Objective	EXP	9.5	52	n.s.	*d_z_* = 0.47 *d_s_* = 0.24
			Empathy (IECA)	Objective	EXP	9.5	52	n.s.	*d_z_* = 0.62 *d_s_* = 0.15
Rickard et al. (2012), Study 2	Enhanced music lessons	20	Aggression Questionnaire	Self-report	EXP	10.9	69	n.s.^c^	*d_z_* = −1.59 *d_s_* = −1.89
Schellenberg et al. (2015)	Group music lessons	40	Prosocial behavior (modified version of SBQ)	Self-report	Q-EXP	8.7	84	+/n.s.^d^	—^b^
			Sympathy (CSS)	Self-report	Q-EXP	8.7	84	+/n.s.^d^	—^b^
Openness									
Ritblatt et al. (2013)	School-readiness music program	28	Approach to Learning (KRS)	Teacher-rated	Q-EXP	4	102	+	*d_z_* = 0.63 *d_s_* = −0.10
Conscientiousness									
Metsäpelto et al. (2012)	Performing arts	156	Working skills (i.e., persistence, concentration, and carefulness)	Teacher-rated	Q-EXP	9.5	177	+	—^b^
Ritblatt et al. (2013)	School-readiness music program	28	School Routines and Work Habits (KRS)	Teacher-rated	Q-EXP	4	102	n.s.	—^b^
Self-esteem									
Costa-Giomi (2004)	Piano lessons	156	Self-esteem (CSEI)	Self-report	Q-EXP	9	80	n.s.	—^b^
Knox Anderson et al. (2007)	Group-based instrumental music training	4	Self-esteem (CFSEI-3)	Self-report	Q-EXP	12.6	44	n.s.	—^b^
Legette (1994, Study 1)	Computer-assisted keyboard program	35	Self-concept (Piers-Harris)	Self-report	Q-EXP	10	97	n.s.	*d_z_* = −0.76
Legette (1994, Study 2)	Computer-assisted keyboard program	15	Self-concept (Piers-Harris)	Self-report	Q-EXP	9.5	141	n.s.	*d_z_* = 0.25
Rickard et al. (2012), Study 1	Enhanced music lessons	22	Self-esteem (CFSEI-3)	Self-report	Q-EXP	12.7	111	n.s.	*d_z_* = −0.45
Rickard et al. (2012), Study 2	Enhanced music lessons	20	Self-esteem (RSE)	Self-report	EXP	10.9	69	n.s.^c^	*d_z_* = 0.19 *d_s_* = −0.31
Rickard et al. (2013; younger cohort)	Enhanced music lessons	104	Self-esteem (CFSEI-3)	Self-report	Q-EXP	6	68	n.s.^e^	*d_z_* = 0.20
Rickard et al. (2013; older cohort)	Enhanced music lessons	104	Global Self esteem (SCFSEI)	Self-report	Q-EXP	9	99	+/−^f^	—^b^
Roy et al. (2015)	School-band program	130	Self-esteem (RSE)	Self-report	Q-EXP	13.5	31	n.s.	—^b^
Zimmermann (2002)	School-band program	39	Self-concept (Piers-Harris)	Self-report	Q-EXP	10	76	n.s.	—^b^
Blended									
Knox Anderson et al. (2007)	Group-based instrumental music training	4	Anger expression (STAXI)	Self-report	Q-EXP	12.6	44	n.s.	—^b^
Metsäpelto et al. (2012)	Music activities	156	Adaptive behavior (TR-MPNI)	Teacher-rated	Q-EXP	9.5	177	+	—^b^
			Externalizing problems (TR-MPNI)	Teacher-rated	Q-EXP	9.5	177	n.s.	—^b^
Portowitz et al. (2009)	Enhanced music lessons	104	Self-concept (TSCS)	Self-report	Q-EXP	8	81	n.s.	*d_z_* = 0.48 *d_s_* = 0.30
Rickard et al. (2012), Study 2	Enhanced music lessons	20	Social skills (SSRS)	Self-report	EXP	10.9	69	n.s.^c^	*d_z_* = 0.00 *d_s_* = −0.22
				Parent-rated	EXP	10.9	69	n.s.	*d_z_* = 0.30 *d_s_* = −0.79
Rickard et al. (2013; younger cohort)	Enhanced music lessons	104	Social skills (SSRS)	Teacher-rated	Q-EXP	6	97	n.s.	—^b^
Rickard et al. (2013; older cohort)	Enhanced music lessons	104	Social skills (SSRS)	Teacher-rated	Q-EXP	9	67	n.s.	—^b^
Ritblatt et al. (2013)	School-readiness music program	28	Social skills (PKBS-2)	Teacher-rated	Q-EXP	4	102	+	*d_z_* = 0.60 *d_s_* = 0.25
				Parent-rated	Q-EXP	4	102	n.s.	—^b^
Roy et al. (2015)	School-band program	130	Promotion focus (GRFM)	Self-report	Q-EXP	13.5	31	+	—^b^

Note: Cohen’s *d_z_* was calculated directly from the *t* statistic divided by the square root of the number of participants. Whenever the *t* statistic was not reported, we calculated the *t* value by dividing the pretest–posttest difference by the standard deviation of the pretest. For experimental studies, we additionally calculated Cohen’s *d_s_* whenever possible (for details, see the Method section). + = positive effect; BASC = Behavioral Assessment System for Children; CFSEI-3 = Culture Free Self-Esteem Inventories Third Edition; CSEI = Coopersmith Self-Esteem Inventories; CSS = Child-Report Sympathy Scale; EXP = experimental design (randomized controlled trial); GRFM = General Regulatory Focus Measure; IECA = Empathy Index for Children and Adolescents; IPIP = International Personality Item Pool; KRS = Kindergarten Readiness Survey; PKBS-2 = Preschool and Kindergarten Behavioral Scale; Q-EXP = quasi-experimental design; RSE = Rosenberg Self-Esteem Scale; SBQ = Social Behavior Questionnaire; STAXI = State Trait Anger Expression Inventory; TEC = Test of emotion comprehension; TSCS = Fitts Tennessee Self Concept; TR-MPNI = Multidimensional Peer Nomination Inventory, Teacher Form.

a *N* refers to the sample sizes of the experimental and control groups combined. ^b^Effect size *d_z_* was not available and it could not be calculated from the results reported in the study. ^c^The reported two-way (Group × Time) mixed-model ANOVA included not only a music group and a control group but also a drama group. ^d^A significant three-way interaction was reported: Group × Time × Level of Performance (low or high). It is unclear whether the two-way interaction (Group × Time) was also significant. ^e^There was no significant effect over the entire 2-year time period. There were significant Group × Time effects in the first year but not in the second year. ^f^There was a significant Time × Group interaction in an ANOVA with three measurement times. In the first year, self-esteem decrease was stronger in the control group than in the music group. In the second year, self-esteem increased more in the control group than in the music group.

**Table 3.** Dance-Education Studies

Study	Intervention	Duration(weeks)	Outcome (measure)	Assessment	Design	*M* Age	*N* ^a^	Effect (test)	Effect size
Emotional Stability									
Lobo et al. (2006)	Creative dance program	8	Internalizing behavior problems (SCBE)	Parent-rated	EXP	4	38	+^b^	*d_z_* = 0.87 *d_s_* = 0.59
				Teacher-rated	EXP	4	38	+^b^	*d_z_* = 0.88 *d_s_* = −0.15
Agreeableness									
Lobo et al. (2006)	Creative dance program	8	Externalizing behavior problems (SCBE)	Parent-rated	EXP	4	38	+^b^	*d_z_* = 0.64 *d_s_* = 0.41
				Teacher-rated	EXP	4	38	+^b^	*d_z_* = 0.40 *d_s_* = −0.12
Pereira et al. (2017)	Educational dance	12	Peer Relations (SSBS-2)	Teacher-rated	Q-EXP	10.5	83	+	*d_z_* = 0.44
			Interpersonal Negotiation (REL-Q)	Self-report	Q-EXP	10.5	90	n.s.	*d_z_* = 0.12
Conscientiousness									
Pereira et al. (2017)	Educational dance	12	Self-management (SSBS-2)	Teacher-rated	Q-EXP	10.5	90	+	*d_z_* = 0.39
			Academic behavior (SSBS-2)	Teacher-rated	Q-EXP	10.5	90	n.s.	*d_z_* = 0.42
Self-esteem									
Seham (1998)	Dance classes	30	Global self-worth (SPPC)	Teacher-rated	Q-EXP	10.5	69	n.s.	*d_z_* = 0.06 *d_s_* = 0.10
Blended									
Pereira et al. (2017)	Educational dance	12	Managing and regulating Emotion scale (ESCQ)	Self-report	Q-EXP	10.5	83	n.s.	*d_z_* = −0.09 *d_s_* = 0.49

Note: Cohen’s *d_z_* was calculated directly from the *t* statistic divided by the square root of the number of participants. Whenever the *t* statistic was not reported, we calculated the *t* value by dividing the pretest–posttest difference by the standard deviation of the pretest. For experimental studies, we additionally calculated Cohen’s *d_s_* whenever possible (for details, see the Method section). + = positive effect; ESCQ = Emotional Skills and Competence Questionnaire; EXP = experimental design (randomized controlled trial); Q-EXP = quasi-experimental design; REL-Q = Relationship Questionnaire; SCBE = Social Competence Behavior Evaluation: Preschool Edition; SSBS-2 = School Social Behavior Scales; SPPC = Self-Perception Profile for Children.

a *N* refers to the sample sizes of the experimental and control groups combined. ^b^In Lobo & Winsler (2006), each outcome was rated by parents and teachers, and they reported the means and standard deviation for teacher and parent ratings for each condition and measurement time separately. Yet for the statistical test, it seems that they aggregated the teacher and parent ratings. Thus, we report for each outcome two effect sizes but only one significance test.

**Table 4.** Visual-Arts-and-Crafts Education Studies

Study	Intervention	Duration (weeks)	Outcome (measure)	Assessment	Design	*M* Age	*N* ^a^	Effect (test)	Effect size
Extraversion									
Goldstein et al. (2012), Study 1; Goldstein et al. (2013), Study 2	Visual arts (vs. acting)	43	Emotional expressivity – positive (BEQ)	Self-report	Q-EXP	8.5	68	n.s.	*d_z_* = 0.29 *d_s_* = −0.15
Emotional stability									
Goldstein et al. (2012), Study 1; Goldstein et al. (2013), Study 2	Visual arts (vs. acting)	43	Emotional expressivity – negative (BEQ)	Self-report	Q-EXP	8.5	68	n.s.	*d_z_* = 0.12 *d_s_* = 0.06
Metsäpelto et al. (2012)	Arts and crafts activities	156	Teacher-rated Internalizing problems (TR-MPNI)	Teacher-rated	Q-EXP	9.5	166	+	—^b^
Agreeableness									
Goldstein et al. (2012), Study 1; Goldstein et al. (2013), Study 2	Visual arts (vs. acting)	43	Theory of mind (Faux Pas Test)	Objective	Q-EXP	8.5	68	n.s.	*d_z_* = 0.47 *d_s_* = 0.01
			Index of Empathy for Children (IECA)	Self-report	Q-EXP	8.5	68	—	*d_z_* = 0.02 *d_s_* = −0.73
Metsäpelto et al. (2012)	Arts and crafts activities	156	Adaptive behavior (TR-MPNI)	Teacher-rated	Q-EXP	9.5	166	+	—^b^
			Externalizing problems (TR-MPNI)	Teacher-rated	Q-EXP	9.5	166	n.s.	—^b^
Conscientiousness									
Metsäpelto et al. (2012)	Arts and crafts activities	156	Working skills (i.e., persistence, concentration, and carefulness)	Teacher-rated	Q-EXP	9.5	166	+	—^b^
Self-esteem									
Catterall et al. (2007)	Visual arts instruction	20-30	General self-concept (self-developed)	Self-report	Q-EXP	9	179	n.s.	—^b^
Rickard et al. (2012), Study 1	Additional art classes	22	Self-esteem (CFSEI-3)	Self-report	Q-EXP	12.7	111	n.s.	*d_z_* = 0.00

Note: Cohen’s *d_z_* was calculated directly from the *t* statistic divided by the square root of the number of participants. Whenever the *t* statistic was not reported, we calculated the *t* value by dividing the pretest–posttest difference by the standard deviation of the pretest. + = positive effect; CFSEI-3 = Culture Free Self-Esteem Inventories Third Edition; EXP = experimental design (randomized controlled trial); Q-EXP = quasi-experimental design; TR-MPNI = Multidimensional Peer Nomination Inventory, Teacher Rating Form

a *N* refers to the sample sizes of the experimental and control groups combined. ^b^Effect size *d_z_* was not available and it could not be calculated from the results reported in the study.

**Table 5.** Mixed-Arts-Education Studies

Study	Intervention	Duration (in weeks)	Outcome (measure)	Assessment	Design	*M* Age	*N* ^a^	Effect (test)	Effect size
Extraversion									
Calero et al. (2017)	Skills development program (incl. arts and theater)	26	Leadership (CPS)	Self-report	Q-EXP	23	322	n.s.	—^b^ *d_s_* = −0.04
Emotional stability									
Wright et al. (2006a, 2006b)	Theater, visual, and media arts	39	Emotional problems	Self-report	Q-EXP	12	366	+	—^b^
Agreeableness									
Ruokonen et al. (2015)	Music, dance, and drama	26	Prosocial behavior (Weir and Duveen scale)	Teacher-rated	Q-EXP	8.5	32	+	—^b^ *d_s_* = 1.22
Wright et al. (2006a, 2006b)	Theater, visual, and media arts	39	Prosocial behavior	Self-report	Q-EXP	12	366	n.s.	—^b^
			Conduct problems	Self-report	Q-EXP	12	366	n.s.	—^b^
Conscientiousness									
Calero et al. (2017)	Skills development program (incl. arts and theater)	26	Order and self-organization (CPS)	Self-report	Q-EXP	23	322	n.s.	—^b^ *d_s_* = 0.14
			Consistency of interest (Grit)	Self-report	Q-EXP	23	322	—	—^b^ *d_s_* = −0.16
			Perseverance of effort (Grit)	Self-report	Q-EXP	23	322	n.s.	—^b^ *d_s_* = 0.06
			Ambition (Grit)	Self-report	Q-EXP	23	322	n.s.	—^b^ *d_s_* = 0.00
Self-esteem									
Calero et al. (2017)	Intensive skills development program (including arts and theater)	26	Self-esteem (CPS)	Self-report	Q-EXP	23	322	n.s.	—^b^ *d_s_* = −0.15
Ruokonen (2018)	Visual art, drama, music, and dance	43	Self-efficacy (NGSE)	Self-report	Q-EXP	16	40	n.s.^c^	—^b^
Wright et al. (2006a, 2006b)	Theater, visual, and media arts	39	Self-esteem	Self-report	Q-EXP	12	366	n.s.	—^b^
Blended									
Ruokonen (2018)	Visual art, drama, music, and dance	43	Social Skills (SSRS-C)	Self-report	Q-EXP	16	40	*+*	—^b^ *d_s_* = 0.07

Note: We were not able to calculate Cohen’s *d_z_* for any of the studies. For experimental studies, we calculated Cohen’s *d_s_* whenever possible (for details, see the Method section). + = positive effect; CPS = Social and Personal Competencies; EXP = experimental design (randomized controlled trial); NGSE = New General Self-Efficacy Scale; Q-EXP = quasi-experimental design.

a *N* refers to the sample sizes of the experimental and control groups combined. ^b^Effect size *d_z_* was not available and it could not be calculated from the results reported in the study. ^c^Ruokonen (2018) did not compute a sum score. Instead, the analysis was conducted for each self-efficacy item separately. They reported a significant effect for only one of the eight items.

The duration of the arts-education programs varied considerably across the selected studies. The shortest program lasted for 4 weeks, whereas the longest extended across a 3-year period. The median duration was 29 weeks. The 36 studies varied with regard to the art form on which the program focused. We categorized the arts activities into five art forms: drama, music, dance, visual arts and crafts, and mixed arts (i.e., a combination of the other art forms). In drama studies, participants engaged in pretend play, dramatic play, improvisational theater, or acting in a film. They played fictitious roles and situations that were improvised, written by students themselves, or provided by a teacher or trainer. In music studies, participants actively played a musical instrument in music lessons or in a band or orchestra. In dance studies, participants attended educational or creative dance programs. In the education-dance programs, participants engaged in guided movement and dance activities. Less guidance was provided in the creative-dance programs. In visual arts-and-crafts studies, participants usually created works of art such as paintings or sculptures. Our mixed-arts category comprised arts education that included more than one of the previous four art activities (drama, music, dance, and visual arts and crafts) or other art forms (e.g., media arts). Most of the arts education was in music (18 studies) or drama (14 studies). Only a few studies used dance (three studies), visual arts and crafts (four studies), or mixed-arts programs (four studies).

The studies varied widely with regard to the outcome measures they investigated, thus rendering them difficult to compare. Therefore, we categorized the different personality variables along the Big Five framework and the domain of self-esteem. To properly categorize the measures, authors M. P. Grosz and C. M. Lechner independently rated the extent to which each measure assessed the six dimensions on the basis of the item content of the measures and the description of the measures provided by the arts-education study. The rating scale ranged from 1 (*does not apply at all*) to 6 (*applies completely*). The interrater reliabilities (i.e., two-way, consistency, average-measures intraclass correlation coefficients) were .79 for extraversion, .76 for emotional stability, .92 for agreeableness, .80 for openness, .87 for conscientiousness, and .90 for self-esteem. We categorized the measures on the basis of these ratings and additionally gathered information (e.g., how the measures correlated with established Big Five measures in past empirical studies) into the six personality domains. Measures assessing affective, cognitive, and behavioral tendencies from more than one personality domain were classified into a blended category (for a similar approach, see Roberts et al., 2017). Most blended measures tended to be associated with extraversion (nine of the eleven blended measures) and agreeableness (eight blended measures). The average ratings, a description and the item content of the measures, and the associations with established Big Five measures can be found in Tables S1 to S7 at https://osf.io/yxqc7/ (for measures that we excluded because they did not assess any of the six personality dimensions, see Table S9 at https://osf.io/8sgmq/). Most outcome measures were in the domain of agreeableness (32 outcomes), followed by emotional stability, self-esteem, and the blended category (20 outcomes each), extraversion (15 outcomes), and conscientiousness (11 outcomes). Only one outcome measure fell into the domain of openness. About half of the outcome measures were assessed via self-report (51%), several were rated by teachers (26%), parents (10%), or observers (7%); and some were objectively measured (i.e., assessed via test or task performance; 6%).^3^

### Effects of arts education on personality

The 36 found studies reported 119 effects. For 64 of the 119 effects (stemming from 21 of the 36 studies), the articles provided enough information to compute the pretest–posttest effect size *d_z_* for the treated group. On the basis of these 64 effect sizes, we estimated the average effect of arts education on personality.^4^ Although many of the effects were not included in this estimation, we found it reassuring that the ratio of positive, negative, and nonsignificant findings was somewhat similar for the 64 effects with available *d_z_* values (48 nonsignificant effects, 13 positive effects, two negative effects, and one partly positive partly negative effect) and the 55 effects without available *d_z_* values (35 nonsignificant effects, 17 positive effects, one negative effect, and two partly positive/partly nonsignificant effects; Tables 1 to 5).

For the estimation of the average effect of arts education on personality, we weighted each effect size by the sample size and the inverse of the number of effect sizes stemming from the sample to account for the dependence among the effect sizes from the same sample. We did not use a random-effects meta-analysis because the test–retest correlation has not been reported for any of the effects *d_z_*, which would have been necessary to estimate the sampling variance. The estimation of the weighted average suggested that arts education induced, on average, moderate personality changes, unweighted average *d_z_* = 0.24; weighted average *d_z_* = 0.22 (1,010 participants). That is, arts education appears to have changed personality, on average, by about one fifth to one fourth of a standard deviation. These average effects are in accordance with previous research and theories indicating that personality traits can be shaped by environmental influences and interventions (e.g., Alan et al., 2019; Golle et al., 2018; Roberts et al., 2017; Wrzus & Roberts, 2017). The weighted average pretest–posttest effect size we found (*d_z_* = 0.22) was smaller than that found in the recent review on the effects of clinical interventions on personality development in adulthood (*d_z_* = 0.37; Roberts et al., 2017). Yet clinical therapies are arguably more intensive than arts education; the latter are usually administered to groups of people rather than individuals.

**Fig. 3.**
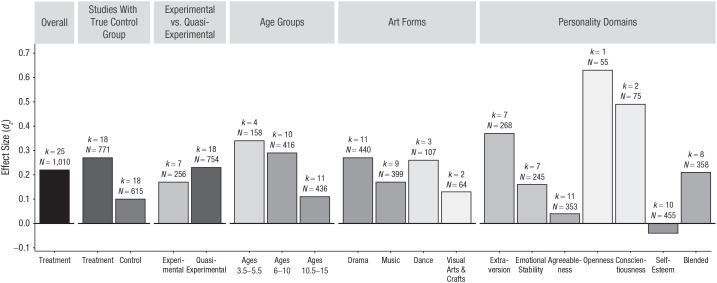
Weighted average effect size *d_z_* for effect of arts education on personality change in various designs, age groups, forms of arts education, and personality domains. The shades of gray indicate the number of participants that the weighted average effect sizes were based upon (darker shades = larger samples). *k* = number of samples; Blended = measures that assessed more than one personality domain.

An alternative explanation for the observed weighted average of *d_z_* = .22 might be that desirable personality change resulted not from participation in arts education but from normative changes in personality (for normative changes, see e.g., Brandes et al., 2020; Orth et al., 2018). To investigate this possibility, we compared the weighted average *d_z_* of the treated groups with the weighted average *d_z_* of the control groups. In this analysis, we included only the 18 samples for which *d_z_* was available for treatment and control group and which had true control groups (i.e., where children in the control group did not receive another type of arts education). The weighted average *d_z_* was 0.27 for the treatment groups (771 participants) and 0.10 for the control groups (615 participants; Fig. 3). This result suggests that the observed personality change in the arts-education groups was not solely the consequence of normative changes.

Another alternative explanation might be that children who were already on a desirable personality-development trajectory were more likely to choose or be selected by teachers or parents for an arts-education program than children on an undesirable trajectory. To probe this possibility, we coded who allocated the participants into the arts education and control groups. For 32 of the 64 effect sizes *d_z_*, participants were randomly assigned (i.e., true experiments). The other 32 effects were coded as quasi-experimental effects. For 14 of the 32 quasi-experimental effects, it was not clear who assigned participants to the experimental and control groups. For 12 quasi-experimental effects, the researchers assigned participants nonrandomly (e.g., students from one school were assigned to the experimental group, whereas students from another school were the control group; Legette, 1994). For three effects, the intention was random assignment but the students were given “some input” (Rickard et al., 2012, Study 1). For one effect, most but not all children were randomly assigned. For one effect, the participants decided themselves in which group to participate. Finally, for one effect, the school decided.

The weighted average effect size *d_z_* was 0.17 for experimental studies (256 participants) and 0.23 for quasi-experimental studies (754 participants; Fig. 3). Furthermore, the difference between the treatment and control groups was smaller among experiments with true control groups (treatment: *d_z_* = 0.17; 256 participants; control: *d_z_* = 0.14; 239 participants) than among quasi-experiments with true control groups (treatment: *d_z_* = 0.32; 515 participants; control: *d_z_* = 0.08; 376 participants). These differences might suggest that selection effects partly accounted for the observed desirable personality change in quasi-experimental studies. Alternatively, the arts education might have led to more desirable personality changes in the quasi-experimental studies than in the experimental studies precisely because the children who received arts education in the quasi-experimental studies might have been more inclined to and thus engaged in the arts education than the children who received arts education in the experimental studies. Future researchers might want to test whether interest in the arts (measured before random assignment to treatment and control group) moderates treatment effects of arts-education interventions.

To investigate whether publication bias is an issue in the literature, we next correlated the sample size with the effect size *d_z_* for the treated groups and found a correlation of −.18 (for a funnel plot, see Fig. S2 https://osf.io/u4mtz/). This indicates that effect sizes for studies with small samples tended to be slightly larger than those for studies with large samples. Publication bias might be one reason for this negative correlation (e.g., Thornton & Lee, 2000). That said, the negative correlation might be caused by factors other than publication bias. For example, the arts education might have been more intensive in studies with smaller samples than in studies with larger samples.

We next partitioned the effects into three age groups to get an idea about whether the effects of arts education on personality are heterogeneous across age. The weighted average *d_z_* was 0.34 in the preschool age group (ages 3.5–5.5; 158 participants undergoing arts education), 0.29 in the elementary school age group (ages 6–10; 416 participants), and 0.11 in the middle school age group (ages 10.5–15; 436 participants). These findings suggest that arts education might be more influential on personality development in early and middle childhood than in preadolescence and early adolescence. Relatedly, adolescents are often less committed to and involved in extracurricular activities than are children, especially if adolescents have the impression that they are not good at these activities (i.e., need for competence) or if their friends are not involved (i.e., need to belong; e.g., Fredricks et al., 2002).

We also calculated the weighted average sample size for each form of arts education separately to get an idea about which art forms are particularly effective. The weighted average *d_z_* was 0.27 for drama education (440 participants), 0.17 for music education (399 participants), 0.26 for dance education (107 participants), and 0.13 for visual arts and crafts education (64 participants; Fig. 3)—we could not compute *d_z_* for any of the mixed-arts studies. In short, drama and dance were on average most effective, music was less effective, and education in visual arts and crafts was least effective. A reason for the effects of drama, music, and dance programs might have been that they provided opportunities for social interactions (Fig. 1). Future researchers might want to test the underlying mechanism that explains why certain forms of arts education are effective. For example, the level of social interactions might be systematically varied to investigate their role.

To get an idea about which personality domains were particularly amenable to arts education, we partitioned the effects into the seven personality categories. The weighted average *d_z_* was 0.37 for extraversion outcomes (268 participants), 0.16 for emotional stability (245 participants), 0.04 for agreeableness (353 participants), 0.49 for conscientiousness (75 participants), −0.04 for self-esteem (455 participants), and 0.21 for blended (358 participants; Fig. 3). For openness, only one effect size (55 participants) was available (*d_z_* = 0.63). These effects are consistent with the proposed paths from drama, music, dance, and visual-arts education to extraversion, conscientiousness, and openness outlined in the introduction (Fig. 1). For example, the repeated social interactions during arts education might have fostered extraversion. Furthermore, the average effect of arts education on conscientiousness is in line with the notion that arts trainings with their behavioral rules demand discipline and self-control and might thus foster conscientiousness. Likewise, research on the effects of homework and vocational training on personality development has suggested that demands for discipline and self-control can lead to increases in conscientiousness (Golle et al., 2018; Göllner et al., 2017). Future research might want to confirm these effects and experimentally manipulate the level of social interactions and required discipline and self-control to test whether these features underlie the effects of arts education on extraversion and conscientiousness. It is noteworthy that arts education does not seem to bolster self-esteem. This is in contrast to, for example, physical-activity interventions in children and adolescents (for a meta-analysis, see Liu et al., 2015). Perhaps the artistic domain matters less for the self-esteem of children and adolescents than do other domains (e.g., sports, physical appearance, and grades).

### Limitations of the reviewed studies and recommendations for future directions

During our review, we noticed several limitations of the existing body of evidence on personality change through arts education. In particular, the conspicuous absence of studies assessing outcomes in the domain of openness is unfortunate, given that openness is the personality domain that is most strongly related to artistic activities, interests, and preferences (e.g., McCrae & Sutin, 2009; Schwaba et al., 2018). Thus, openness to experience might be most strongly affected by arts education (Fig. 1). We think there are two reasons for the lack of studies on openness. Many reviewed studies focused on social, emotional, and working skills. Openness is probably less frequently considered to be a social, emotional, or working skill than are other Big Five traits (e.g., Schwaba et al., 2019). Second, openness is the most controversial personality domain of the Big Five in childhood personality models (Herzhoff et al., 2017; Herzhoff & Tackett, 2012). For example, openness has no equivalent in the four major dimensions of child-temperament models: sociability, negative emotionality, persistence, and activity level (e.g., De Pauw et al., 2009). That said, newer research has demonstrated that openness can be reliably and distinctly measured at least from middle childhood onward (e.g., Herzhoff & Tackett, 2012). Hence, we encourage future arts-education intervention researchers to include outcomes in the domain of openness.

Another limitation of the reviewed literature is that almost none of the studies did a follow-up assessment of the outcome variables to test how enduring the effects of the arts education were. Because personality-trait measures are contaminated with state-related content, only follow-up measurements can reveal whether an intervention actually led to enduring personality-trait change or led only to transient shifts in states (e.g., Roberts et al., 2017). For instance, one of the few arts-education intervention studies with more than two measurement points found a desirable effect of music lessons on self-esteem in the first year. Yet in the second year, this effect was not significant in the younger cohort and was even reversed in the older cohort (Rickard et al., 2013). Thus, the effect observed in the first year might have been due to changes in episodic state self-esteem rather than to changes in trait self-esteem. Future researchers need to test not only the existence but also the durability of the effects of arts education on personality.

Furthermore, the literature on the effectiveness of arts education is characterized by a large degree of heterogeneity, not only in the types of interventions and the age of participants but also in outcome measures. To facilitate the comparability and interpretation of the diverse outcome measures prevalent in the literature, we used the Big Five taxonomy and self-esteem to categorize the outcomes measures into six personality domains. A reason for diverse outcome measures and the lack of direct assessments of the Big Five is certainly that the direct measurement of the Big Five in youth has gained traction only in recent years (e.g., Soto, 2016; Soto & Tackett, 2015). Future researchers will need to confirm our findings with personality measures that more directly and broadly assess the Big Five in children and adolescents. That said, it might also be worthwhile to study the effects of arts education on personality facets and nuances because these specific aspects might be more amenable than broad traits. The results of the primary studies depicted in Tables 1 to 5 might serve as initial evidence for such studies.

Moreover, many of the reviewed studies seem to have had low statistical power. Although most studies did not conduct or report a power analysis and it is thus unclear how high powered they were, several studies did not meet lower-bound recommendations, such as the requirement to have 20 observations per condition (e.g., Simmons et al., 2011). Low statistical power is problematic not only because existing effects will rarely be detected (i.e., high rates of false negatives) but also because flexibility in study design, data collection, and data analysis inflates false-positive rates more strongly in studies with small samples than in studies with large samples (e.g., Simmons et al., 2011).

Finally, we found a negative correlation between sample size and effect size, which might be a consequence of publication bias. We recommend that future researchers use preregistration and the registered-report format to reduce the risk of publication bias (e.g., Chambers, 2013; Thornton & Lee, 2000).

### Conclusion

Collectively, the studies reviewed in the current article suggest that arts education might indeed be a viable means by which to foster desirable personality change. For example, arts-education programs appear to foster extraversion and conscientiousness, which would be in line with the theoretical pathways that we proposed. However, the evidence for the effectiveness of arts education was very limited among the few studies that used true experimental designs. Generally, the reviewed studies were small in number, heterogeneous, and subject to a number of content-related, methodological, and statistical limitations. More research is needed, and these limitations need to be addressed before solid implications for policymaking, educational practice, and personality theories can be drawn. Thus, a main contribution of the current review is to illustrate the lack and limitations of existing evidence on arts education and personality change and to point out promising future directions. In so doing, we hope that our review spurs not only further research but also methodological improvements and thereby paves the way for understanding whether and how arts activities shape the personality of children and adolescents.

## Transparency

*Action Editor:* Richard Lucas

*Editor:* Laura A. King

## Notes

1. We did not include neuroticism into the paths and Figure 1 because we did not find pertinent evidence and theories that would point to potential paths from arts education to neuroticism.

2. We were not able to take into account the correlation between the pretest and posttest when calculating the standard deviation of the difference score because it was not reported in any of the studies.

3. We have also coded whether the item content of the included measures referred mainly to an arts-education context, a school context, or was context free (i.e., referred to behavioral, cognitive, and affective tendencies *in general*). For 89 out of 119 effect sizes, the majority of the items were context-free. For 17 out of 119 effect sizes, the majority of the items referred to a school context. We think it is acceptable that some self-report and teacher-rating items referred to a school context because it might be difficult for children to answer very context-free (abstract) items about themselves, and teachers might know children only from the school context. For none of the 119 effect sizes did the majority of the items refer to an arts-education context. For five out of 119 effect sizes, the item content was a mixture between context free and school context (i.e., about half of the items referred to a school context and the other half of the items were context free). Furthermore, for eight out of 119 effect sizes, the item content was not reported and thus could not be coded by us.

4. We did not include postintervention difference effect size *d_s_* in this estimation because *d_s_* and *d_z_* are only comparable under specific circumstances (e.g., Lakens, 2013).
